# Experiences of women with type 2 diabetes during the pre‐pregnancy, pregnancy and postpartum periods: A systematic review of qualitative studies

**DOI:** 10.1111/dme.70094

**Published:** 2025-07-03

**Authors:** Lily Hopkins, Noreen O'Leary, Aileen Burton, Eleanor Dyer, Angela C. Flynn, Sowmiya Gunabalasingam, Nicola Heslehurst, Artemis Kyrka, Rivka Lebrett, Claire L. Meek, Danielle A. J. M. Schoenaker, Sara L. White, Rita Forde

**Affiliations:** ^1^ Department of Non‐Communicable Disease Epidemiology, Faculty of Epidemiology and Population Health London School of Hygiene and Tropical Medicine London UK; ^2^ School of Population Health Royal College of Surgeons in Ireland Dublin Ireland; ^3^ School of Nursing and Midwifery University College Cork Cork Ireland; ^4^ Population Health Sciences Institute, Newcastle University Newcastle upon Tyne UK; ^5^ Department of Nutritional Sciences, School of Life Course and Population Sciences King's College London London UK; ^6^ Department of Women and Children's Health, School of Life Course and Population Sciences King's College London London UK; ^7^ Clalit Health Services Rehovot Israel; ^8^ Salford Royal Hospital, North West of UK Foundation School Salford UK; ^9^ Leicester Diabetes Centre and Leicester NIHR Biomedical Research Centre University of Leicester, Leicester General Hospital Leicester UK; ^10^ School of Human Development and Health, Faculty of Medicine University of Southampton Southampton UK; ^11^ MRC Lifecourse Epidemiology Centre University of Southampton Southampton UK; ^12^ NIHR Southampton Biomedical Research Centre University of Southampton and University Hospital Southampton NHS Foundation Trust Southampton UK; ^13^ Department of Diabetes and Endocrinology Guy's and St Thomas' Hospital NHS Foundation Trust London UK; ^14^ Faculty of Nursing, Midwifery and Palliative Care King's College London London UK

**Keywords:** pre‐pregnancy, pregnancy, postpartum, qualitative methods, systematic reviews, type 2 diabetes

## Abstract

**Aim:**

To explore the experiences of women with type 2 diabetes prior to, during and after pregnancy.

**Methods:**

Six databases were systematically searched in September 2023. Qualitative studies involving women of childbearing age living with type 2 diabetes in the pre‐pregnancy, pregnancy and postpartum periods were included. A thematic synthesis was conducted to inductively generate themes related to the experiences of women with type 2 diabetes. Descriptive themes were mapped to the Socio‐Ecological Model to generate analytical themes relating to the personal, interpersonal and organisational level impacts that underlie women's experiences. Study quality was assessed using the CASP checklist for qualitative studies.

**Results:**

Eleven papers from seven countries met the inclusion criteria. Four themes containing six subthemes were generated. At a personal level, women prioritised their desire to plan a family over *‘family planning’*; however, once pregnant they were fearful for the future health of their children. At the interpersonal level, women reported that their self‐efficacy was influenced by family and socio‐cultural factors which often lacked diabetes‐specific information. At the organisational level, women described a fragmented healthcare system and felt that healthcare professionals' unfamiliarity with the reproductive health needs of women with type 2 diabetes resulted in ineffective integration into their diabetes care.

**Conclusion:**

The provision of appropriate care to women with type 2 diabetes prior to and during pregnancy is inadequate, leaving them vulnerable to increased health risks and emotional distress. Innovative ways are needed to embed reproductive healthcare into mainstream diabetes management for women with type 2 diabetes prior to and post‐pregnancy.

**What's new?**

- Reproductive healthcare experiences among women with type 2 diabetes have remained largely unchanged over the past decade, despite the growing prevalence of this group.
- Women with type 2 diabetes continue to report a lack of understanding about the management of diabetes before and during pregnancy, contributed to by fragmented and inconsistent care.
- Healthcare professionals need to be supported to engage in proactive and supportive discussions about pregnancy with women with type 2 diabetes. Consideration must be given to the integration of the management of mental well‐being and involve women's social support systems.

## 1 INTRODUCTION

The number of pregnancies complicated by type 2 diabetes is increasing.^1^ In the United Kingdom, this group represents the majority of pregnancies among individuals with pre‐existing diabetes, currently accounting for 55% of such pregnancies.^1^, ^2^ These pregnancies are considered high risk, as women with type 2 diabetes have additional risk factors for serious adverse pregnancy outcomes.^2^, ^3^ The proportion of congenital anomalies, stillbirths and neonatal deaths is now higher in the infants of women with type 2 diabetes compared to those with type 1 diabetes.^2^ Additionally, compared to women with type 1 diabetes, women living with type 2 diabetes tend to be older (mean age of 34 years at delivery compared to mean age of 30 years), have a higher body mass index (BMI) and have more metabolic comorbidities requiring concomitant medications.^2^, ^3^, ^4^ Women with type 2 diabetes are also more likely to belong to minoritised ethnic groups and live in the highest areas of socio‐economic deprivation when compared to those with type 1 diabetes.^1^, ^2^, ^4^, ^5^ Such characteristics are associated with poorer pregnancy outcomes independent of diabetes.^4^, ^6^

A recent UK national audit reported suboptimal care prior to pregnancy among women with type 2 diabetes, as approximately one in seven were taking ACE inhibitors and/or statins (13%), as well as other potentially harmful diabetes therapies (7%) when presenting for care when pregnant.^1^ Fewer than one in four women with type 2 diabetes were taking the recommended high dose of folic acid before pregnancy.^1^ The difference between preparedness for a diabetes pregnancy is widening between the groups, as women with type 1 diabetes are almost twice as likely to meet the composite NICE pre‐pregnancy care criteria^7^ compared to those with type 2 diabetes, 17.6% versus 9.5%, respectively.^1^

A review and meta‐synthesis was conducted in 2016 to explore the experiences of pre‐pregnancy care for women with type 2 diabetes.^8^ This review identified that the uptake of pre‐pregnancy care was informed by women's personal orientation to their reproductive needs, their negative interactions with healthcare professionals and healthcare system‐level factors. A lack of awareness about the pre‐pregnancy care needs for this group, a lack of communication between healthcare professionals and women with type 2 diabetes, and the absence of systemic processes to incorporate pre‐pregnancy care into their routine diabetes care were also identified.^8^ These data suggest that planning for pregnancy is not a priority for women themselves, their healthcare professionals or the healthcare system, despite the evidence and clinical guidelines in support of this care.

Beyond pre‐pregnancy care, little is known about the experiences of women with type 2 diabetes during pregnancy and postpartum. A systematic review on the topic of women's experiences of diabetes and diabetes management in pregnancy published in 2014 found only four eligible studies with participants specified as having type 2 diabetes, out of 22 included studies, and did not report specific findings for women with type 2 diabetes.^9^ Given the differences in the characteristics and care experiences of women with type 2 diabetes, it is important to independently explore their experiences of pregnancy so that their needs can be understood. Furthermore, to the authors knowledge, no previous reviews have been published on the postpartum experiences of women with type 2 diabetes.

The majority of women with type 2 diabetes during pregnancy are classified as having early‐onset diabetes. There is growing recognition of this form of diabetes and its association with a higher incidence of complications compared to diagnoses occurring later in life. This study aimed to expand upon insights gained from a previous review of pre‐pregnancy care by broadening the scope to encompass the entire pregnancy journey and focusing exclusively on women the type 2 diabetes, not including healthcare professionals' perspectives unlike the previous review. Specifically, we explored women's experiences of care before, during and after pregnancy.

## 2 METHODS

This review was conducted as part of a broad programme of systematic reviews which aimed to explore optimal management for women with type 2 diabetes pre‐pregnancy, during and post‐pregnancy, including interventional, observational and qualitative study designs. A protocol for a broad programme of related systematic reviews was registered in the PROSPERO database (CRD42021292405). The search for the programme of systematic reviews was designed to be highly sensitive, rather than specific to certain study designs. Database searching, title and abstract, and full‐text screening was conducted for the broader programme of systematic reviews before identified articles were divided according to their study design. The current review focuses on qualitative studies conducted with women with type 2 diabetes pre‐pregnancy, during and post‐pregnancy with the specific aim of exploring their experiences during these time periods. This review is reported according to PRISMA guidelines.^10^

### 2.1 Inclusion and exclusion criteria

The eligibility criteria for the broader programme of reviews was developed in reference to the population, exposure, comparison, outcomes and study designs of interest (PECOS) framework. The eligibility criteria for the broad package of reviews have been described in more detail previously.^11^ Studies were included in this review if they incorporated: (1) women of childbearing age living with type 2 diabetes in the pre‐pregnancy, pregnancy and postpartum periods; and (2) used a qualitative study design.

Studies were excluded if they were (1) non‐qualitative designs; (2) non‐primary data studies such as reviews; (3) grey literature, including conference abstracts; (4) study populations comprised of women living with type 2 diabetes not in pre‐pregnancy, pregnancy or postpartum periods; (5) studies not published in the English language; (6) studies that did not report disaggregated data for the type 2 diabetes population if other groups were included; and (7) studies conducted before 2000, to reflect the considerable changes in type 2 diabetes pregnancy prevalence and management. This review aimed to build on the findings from a previous meta‐synthesis on the experiences of pre‐pregnancy care for women with type 2 diabetes^8^ and expand the focus to account for the lack of previous reviews on experiences of women with type 2 diabetes during pregnancy and postpartum. Therefore, the qualitative studies included in the previous pre‐pregnancy care review were excluded from the current study.

### 2.2 Literature search and study selection

For full details of the database searches, see Data S1. MEDLINE, EMBASE, CINAHL, PsychINFO, ASSIA, and the Cochrane library databases were searched using a combination of Medical Subject Headings (MeSH) and keywords in February 2022, with an updated search conducted in September 2023. Search terms were based on five main concepts: pre‐pregnancy, pregnancy, inter‐conception, postpartum and type 2 diabetes. Boolean combinations were used to connect search terms and were adjusted according to the requirements of each database. Searches were limited to human studies. Reference list searching was conducted manually and citation searching was conducted using Google Scholar on included studies.

### 2.3 Study selection

Identified citations were transferred to EndNote version 20 and duplicates removed before citations were uploaded to Covidence for title and abstract screening against eligibility criteria. All titles and abstracts were screened by two independent reviewers. Articles that clearly did not meet eligibility criteria were excluded. The full texts of remaining articles were reviewed independently by two authors. At all stages of study selection, disagreements between reviewers were resolved through consensus discussion among the study team.

### 2.4 Data extraction

A standardised data extraction template was created which included title, author, publication date, study focus, country of study and qualitative data. Data extraction was performed by two authors independently (RF and LH). Any disagreements were resolved through discussion with the wider study team.

We extracted any quotes from included studies that were identified as being (1) related to women with type 2 diabetes and (2) contextually related to pre‐pregnancy, pregnancy or postpartum periods. Associated themes and data codes were extracted to ensure context was maintained throughout data synthesis, for example, by ensuring quotes were attributed to the correct time periods (e.g. pre‐pregnancy vs. postpartum).

### 2.5 Data synthesis

A thematic synthesis was conducted to combine the findings of the identified qualitative studies in line with the methods outlined by Thomas and Harden.^12^ This involved three main steps: (1) descriptively coding the extracted quotes using an inductive approach; (2) inductively developing initial descriptive themes; and (3) generating analytical themes.^12^ Two reviewers (RF and LH) independently coded the quotes which were then agreed through consensus discussion. The descriptive themes were developed collaboratively (RF, LH, NOL, AB, ED) and agreed through consensus discussion between the wider team. Descriptive themes were initially generated by two members of the study team (LH and RF) by labelling the included quotes with descriptive codes. Descriptive codes were then grouped into common descriptive themes through discussion via an iterative process with the wider study team (RF, LH, NOL, AB, ED).

Descriptive themes were then mapped to the Socio‐Ecological Model^13^ to generate analytical themes to structure the analysis. This model was chosen due to its focus on the interplay between individual, societal and community influences on health, which was reflected in the descriptive themes from the data.^13^ The *personal* level is the individual characteristics of the person as well as their prior experiences. The *interpersonal* level is the group that surrounds an individual, such as family. The *organisational* level considers the structured communities to which groups of individuals belong, such as services within the healthcare systems.^13^ Additionally, external stakeholders are those who can influence the interpersonal level through their interactions. For example, if emphasis is placed on an aspect of care such as the need to meet specific glucose targets during pregnancy, then this can result in the individual altering their attitude or behaviour, either positively or negatively, in response to that influence.

### 2.6 Quality assessment

The Critical Appraisal Skills Programme (CASP) checklist for qualitative studies^14^ was used to critically appraise the quality of the included studies. Critical appraisal was conducted by two reviewers (RF and LH) with disagreements resolved through consensus opinion. Results of the quality assessment were not used as inclusion or exclusion criteria for this review.

## 3 FINDINGS

**FIGURE 1:**
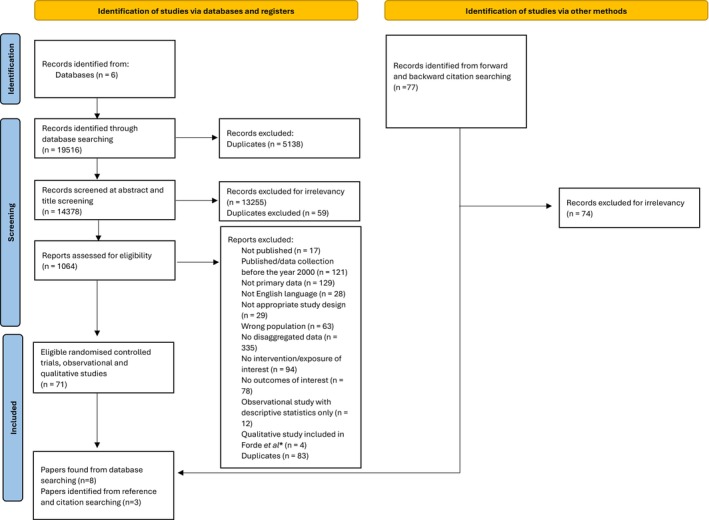
PRISMA flow diagram. *Reference: Forde R, Patelarou E, Forbes A. The experiences of pre‐pregnancy care for women with type 2 diabetes mellitus: a meta‐synthesis. Int J Womens Health. 2016 Dec; Volume 8:691–703.^8^

Database searching for the broad package of systematic reviews identified a total of 19,516 studies (Figure 1). After duplicates were removed (*n* = 5138), 14,378 titles and abstracts and 1064 full texts were screened against eligibility criteria. After removal of those studies eligible for inclusion in the interventional or observational reviews, eight papers met criteria for inclusion in this qualitative review. A further three papers were identified from reference and citation searching. In total, 11 papers met the inclusion criteria for this review, exploring the experiences and views of 162 women living with type 2 diabetes.

### 3.1 Characteristics of included studies

Of the included studies, three were conducted in the United Kingdom,^15^, ^16^, ^17^ three in the United States of America,^18^, ^19^, ^20^ and one each in Turkey and the United Kingdom,^21^ Australia,^22^ Canada,^23^ Malaysia^24^ and Zimbabwe.^25^ Eight studies focused on care prior to pregnancy^15^, ^16^, ^17^, ^18^, ^19^, ^20^, ^22^, ^24^ and two on pregnancy care.^23^, ^25^ One study focused on the experiences of women across their reproductive years.^21^ While none of the studies specifically focused on women's experiences during the postpartum period, some explored pregnant women's thoughts and concerns around this period. Three studies comprised of women with type 2 diabetes only,^19^, ^21^, ^24^ two studies involved women with type 2 diabetes and healthcare professionals,^15^, ^16^ and the remaining six included women with both type 1 and type 2 diabetes,^17^, ^18^, ^20^, ^22^, ^23^, ^25^ of which one also included women with GDM^25^ and another included partners.^22^ An overview of all the included studies is presented in Data S2. Quality assessment scores using the CASP tool categorised the included studies as medium to high quality. Quality assessment identified that most studies reported explicit research questions and had an appropriate methodology to answer these research questions. However, many studies did not explicitly consider the relationship between the researchers and the participants.

### 3.2 Applications of the Socio‐Ecological Model

**FIGURE 2:**
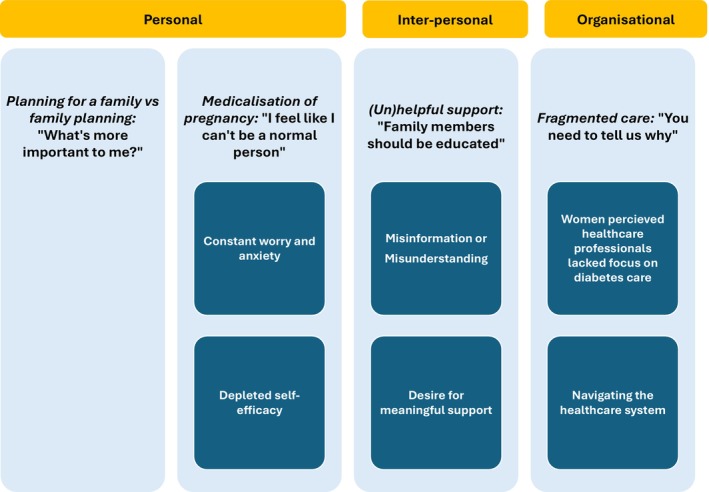
Descriptive themes from the data according to the Socio‐Ecological Model.

The personal, interpersonal and organisational level influences of the Socio‐Ecological Model are reflected in the descriptive themes from the data (Figure 2). At the personal level, two themes were generated portraying the discord of living with type 2 diabetes and the need for specific care prior to and during pregnancy: ‘Planning for a family vs ‘*family planning*’ and ‘Medicalisation of pregnancy’.’ Within the ‘Medicalisation of pregnancy’ theme, two subthemes were identified: ‘Constant worry and anxiety’ and ‘Depleted self‐efficacy’. At the interpersonal level, one theme was identified, ‘(Un)helpful support’, with two subthemes depicting the impact of external stakeholder interactions and support: ‘Support is based on misinformation or misunderstanding’ and ‘Desire for meaningful support’. Finally, at the organisational level, one theme was identified, ‘Fragmented care’, with two subthemes illustrating healthcare system‐level influences on care delivery for women living with type 2 diabetes: ‘Women perceived healthcare professionals lacked focus on diabetes care’ and ‘Navigating the healthcare system’. See Tables 1, 2, 3 for quotes associated with each descriptive theme.

**TABLE 1:** Socio‐Ecological Model: personal level—descriptive themes, subthemes and associated quotes.

Themes	Subthemes	Associated quotes
Planning for a family vs. family planning	‘What's more important to me?’	*‘What's more important to me right now? Is it to have a child, or to have my sugar level at the correct level? I'm just gonna try and conceive anyway.’* ^15^
		*‘I said*—*in my mind, I do not tell them* [*healthcare professionals*]—*I said, instead of doing that*[*commencing diabetes medication*] *right now, let me just have a go for a baby.’* ^15^
		*“I think looking back I would've liked somebody to say something. But I also don't think that I would've been receptive to it.”* ^18^
Medicalisation of pregnancy: ‘I feel like I can't be a normal person’	*Constant worry and anxiety*	*‘You feel guilty when your blood sugar is high… you're like, ‘What important body part is being formed right now? And am I ruining it?”* ^23^
		*‘If it is up to me, I want a second child, but my husband does not want it because of my diabetes… My husband says “you have diabetes, what if our second child becomes like our first child who was born too early* [*36 weeks of pregnancy*],*” he is afraid too, I am frankly scared.’* ^21^
	*Depleted self‐efficacy*	*‘*“*Well I can't do much of exercise*. … *I mean I have got a back problem as well like slipped disc and err exercise, I used to go to the gym, and I used to go swimming as well but to be honest I don't do anything now”…*This participant in a pre‐pregnancy care study—went on to explain*: “*[*Be*]*cause of my health I sometimes feel dizzy at times, I just feel weak in myself* […] *so I don't go out much*.”’^17^
		*‘…My husband is not formally employed. He just does casual work which he sometimes fails to find. As a result, our family income is not constant and sometimes I fail to buy insulin. Even though I have my glucometer that I got from ZDA, I cannot afford to buy test strips to test myself.’* ^25^
		*‘You have to be prepared for everything. You gonna have to modify because… day one week one, may not be the same as week 37 or 38 or 39 or 40 tryin’ to deliver. So, like I tell people, nowhere in the rulebook does it say you can't cry, it just says you can't quit. That's, you have to be in control. ‘Cause I tell diabetes every day, you will not beat me. We can fight all day, but you will not beat me.’* ^20^

**TABLE 2:** Socio‐Ecological Model: interpersonal level—descriptive themes, subthemes and associated quotes.

Themes	Subthemes	Associated quotes
(Un)helpful support: ‘Family members should be educated’	*Support is based in misinformation or misunderstanding*	*‘Family members should support women with sugar. They should be educated on diabetes in pregnancy. It is not a demon but just a disease like any other. If they are taught about it they will not force us to get help from faith healers and traditional healers but will actually encourage us to take our medications and eat the correct foods.’* ^25^
		*“I have not used contraception for a long time. Since my last childbirth in 2009 until now, I have not used any contraception. No effect (getting pregnant) at all …*. *because my mother gave me some traditional medicine, to stop me from conceiving again, seeing that I am sick.”* ^24^
	*Desire for meaningful support*	*‘I love my best friend, but I need a diabetic best friend to go through this with me … so I know that there's somebody else in my corner, that's actually going through this and understands everything….’* ^23^
		*‘Facebook pages are great…if it wasn't for that I wouldn't know…so I found it really helpful’* ^22^
		*‘It is very difficult to stick with your diet when you are at home with other people especially in‐laws. I need to prepare my own meals that do not have cooking oil and sugar but they will think I am cooking more delicious food that I do not want to give them. Sometimes they will say that I do not want to mix with them or I am greedy. My husband hears all this but he does not say anything. As a result I just eat what has been cooked for everyone to avoid problems.’* ^25^

**TABLE 3:** Socio‐Ecological Model: organisational level—descriptive themes, subthemes and associated quotes.

Themes	Subthemes	Associated quotes
Fragmented care: ‘You need to tell us why’	Women perceived healthcare professionals lacked focus on diabetes care	*‘If the nurses doing the annual reviews, has a type 2 woman of child‐rearing age they may wish just to drop pre‐pregnancy care into the conversation; you know, when they ask you if you're depressed and having suicidal thoughts, which they seem to ask you every single year, because I know they have to.’* ^15^
		*‘You need to tell us why, why it is important, not just, do this, … I would have tried harder if I'd known beforehand* [*current pregnancy*] *but now I have to wait and see what I've done’* ^16^
		*‘I don't know if the medical practitioners assume that diabetes is only affecting mainly the older people such that there's no need of giving information about diabetes and pregnancy. They kind of don't pay attention to that.’* ^19^
		*‘When I contacted him* [*GP*] *and said, “I need a prescription for folic acid, 5 mg,” he went, “Oh, gosh, why do you need that much?” And I was, like, “Well, because I'm diabetic.” And he actually had to go away and look it up and he called me back and he said, “I am very sorry, you are quite right”, and diabetes is one of his areas that he's interested in.’* ^15^
		*‘I went to a clinic regarding my diabetes and they asked me if I wanted to get pregnant. I told them ‘Yes'* … *and they told me that I should wait until I get my sugar under control.’’* ^18^
	Navigating the healthcare system	*‘One of the nurses in the eye clinic asked me about it, so I said I wanted to have one more child. She put me forward for the preconception, but I wouldn't have known without her.’* ^15^
		*‘Once, the GP did say that if I become pregnant there will be is a special department or clinic that will look after me, but nothing about before.’* ^15^
		*‘There should be a special diabetic care unit for pregnant women with sugar… We will get to be given comprehensive health education and we understand exactly what we should do to keep our blood sugar low.’* ^25^
		*‘It's been a year… they've took me off the list for diabetes, and nobody has been calling me in* […] *and I didn't know, no blood tests nothing for about a year and a half, then I went back to my GP, and I says I need to referred, either you guys check me or send me back to the diabetic clinic because I didn't know what's going on…’* ^17^

### 3.3 Socio‐Ecological Model: personal level

#### 3.3.1 Theme 1: planning for a family vs. *‘family planning’*: ‘what's more important to me?’

For women living with type 2 diabetes, a tension exists between the advice they receive from healthcare professionals around family planning, such as using contraception to avoid or delay pregnancy until their diabetes has been optimised and pursuing their desired reproductive goals. Some women reported prioritising their wish to have a baby over managing their diabetes, indicating that their reproductive desires often took precedence over the potential health risks associated with pregnancy and type 2 diabetes. This prioritisation frequently conflicted with the medical guidance they received, reflecting a complex interplay between personal reproductive goals and the clinical management of their diabetes. Women often lacked knowledge about the relationship between type 2 diabetes and pregnancy, leading to difficulty understanding the importance of the advice given. These perspectives and priorities influenced some women's decisions regarding contraception and diabetes medication, while also intensifying anxiety related to fertility (Table 1).

#### 3.3.2 Theme 2: medicalisation of pregnancy: ‘I feel like I can't be a normal person’

Women reported type 2 diabetes negatively impacting their pre‐pregnancy and pregnancy experiences mainly due to the heightened medicalisation and intensified focus on risk. They described the constant worry, anxiety, and the amplified management burden of diabetes. Many women described feeling powerless with diminished agency and self‐efficacy during this life stage. This theme is represented in the following two subthemes (Table 1).

##### Constant worry and anxiety

Women reported a range of emotions related to living with diabetes prior to and during pregnancy that significantly shaped their experiences. A major concern was the potential impact of diabetes and specifically glucose levels on fetal development.

There were also concerns about the impact of diabetes on the birthing process, as such a diagnosis resulted in the elimination of choice regarding some birthing options due to the ‘high‐risk pregnancy’ status. Many women were concerned their baby would be born with diabetes or experience complications such as neonatal hypoglycaemia. While for others, their worry and anxiety centred about their own post‐natal health.

Some women internalised their partners' worry and anxiety about the impact of type 2 diabetes influencing decision making about a future pregnancy.

##### Depleted self‐efficacy

Women's perception of their ability to achieve their diabetes‐related goals was variable; many described feeling powerless and lacking in agency and self‐efficacy throughout their pre‐pregnancy and pregnancy experiences. Self‐efficacy was shaped by physical, cultural, financial and emotional influences.

Some women reported being physically unable to engage in exercise due to health‐related issues like a back injury. Dietary advice often failed to consider personal preferences or culturally appropriate foods, making it difficult to follow. For some women, financial constraints further limited access to recommended foods. In study settings without subsidised healthcare, financial constraints also limited access to glucose monitoring supplies and medications which negatively affected their self‐management.

Emotionally, the burden of living with type 2 diabetes during pregnancy increased stress and negatively impacted their overall well‐being. Women struggled to balance physical changes and diabetes management with their mental health.

Conversely, some women expressed a strong determination to manage their diabetes, viewing pregnancy as a motivator for long‐term health improvements. For this group, achieving physical health goals was considered integral to overall well‐being and future pregnancy outcomes.

### 3.4 Socio‐Ecological Model: interpersonal level

#### 3.4.1 Theme 3: (Un)helpful support: ‘family members should be educated’

At the interpersonal level of the Socio‐Ecological Model, the impact of interactions with family, friends and peers was influential, as women sought support. However, while well intentioned, these interactions were not always based on accurate information and, in the context of a type 2 diabetes pregnancy, had the potential to be harmful. Women expressed their desire for meaningful support from those who understood them. This theme comprises of two subthemes: *Support is based on misinformation or misunderstanding* and *Desire for meaningful support* (Table 2).

##### Support is based on misinformation or misunderstanding

Family members and friends were often influential in relation to decision making about contraception use and family planning. The basis for such information and advice was grounded in their own personal experiences and beliefs, rather than factual or diabetes‐specific guidance. These interactions with family and friends had the potential for misinformation or misunderstanding particularly about contraception.

##### Desire for meaningful support

Women expressed a need for more meaningful support, particularly from peers, so they could share experiences with those who understood their challenges. The lack of peer support was described as ‘*a real missed opportunity*’, with women actively seeking others, including via social media. Women voiced the importance of meaningful family support, particularly regarding dietary requirements. However, a lack of understanding among family members often made it more difficult to follow the recommended guidance.

### 3.5 Socio‐Ecological Model: organisation level

#### 3.5.1 Theme 4: fragmented care: ‘you need to tell us why’

At the organisational level of the Socio‐Ecological Model, care delivery and receipt were influenced by factors such as policies, practices and procedures that were both locally and nationally driven. Women with type 2 diabetes reported a dissonance between their diabetes care and their reproductive health care needs, resulting in fragmented care. This theme comprises two subthemes: *Women perceived healthcare professionals lacked focus on diabetes care* and *Navigating the healthcare system* (Table 3).

##### Women perceived healthcare professionals lacked focus on diabetes care

Interactions with healthcare professionals were often shaped by organisational level influences. Women perceived that care delivery was heavily influenced by processes such as renumeration, structured around achieving targets that did not include reproductive healthcare for women with type 2 diabetes. When reproductive healthcare was included, women reported information being conveyed in a manner that was directive, lacking explanation or was ambiguous, and they could not relate to it. Instead, women wanted to have the relationship between diabetes and pregnancy explained in a way they could understand.

As their type 2 diabetes was routinely managed in primary care, the absence of discussions regarding diabetes and pregnancy led some women to question whether healthcare acknowledged their reproductive potential or the trend toward earlier age of onset. Concerns were also expressed about healthcare professionals' knowledge, as access to specific pre‐pregnancy care was delayed by requirements to attain specific target glucose levels before a referral would be made.

##### Navigating the healthcare system

A lack of clarity about the clinical care pathway for women with type 2 diabetes prior to, during or post‐pregnancy was reported. Access to specific care was often by chance and depended on who the woman met, rather than such information being included routinely.

At all levels of the healthcare system, transitioning between services posed challenges due to both a lack of awareness of specialist clinics and arbitrary requirements imposed by healthcare professionals before a referral would be facilitated. Many women reported a lack of awareness about the availability of pre‐pregnancy care clinics. It was also evident that not all women had access to or were aware of diabetes‐specific antenatal care. While for others, the transition from antenatal care back to routine type 2 diabetes care following the birth of their baby was disjointed and risked women being removed from routine recalls as it was perceived they had missed appointments.

## 4 DISCUSSION

This review sought to explore the experiences of women living with type 2 diabetes before, during and after pregnancy, to better understand their care needs. We also aimed to build upon the findings of the previous meta‐synthesis on pre‐pregnancy care in 2016, which included studies published between 2010 and 2015.^8^ We identified only 11 studies (published between 2017 and 2023) meeting the inclusion criteria for this review; a relatively small number given the rapidly increasing prevalence of type 2 diabetes in women of reproductive age.^1^ Furthermore, none of the identified studies focused on the experiences of women with type 2 diabetes in the postpartum period.

There is limited research in this area which reflects inequality in the attention women with type 2 diabetes receive before, during and after pregnancy in both research and standard clinical practice. This concurs with previous reviews of experiences of and interventions to enhance pre‐pregnancy care for women with type 2 diabetes.^8^, ^26^ Indeed, the current review suggests that little has changed for women with type 2 diabetes in the last decade, despite the increasing prevalence of this group.^1^, ^2^ Women continue to report care which is inconsistently delivered and fragmented from their routine interactions. Advice is often delivered ambiguously and without consideration of women's needs or perspectives. Therefore, it is becoming increasingly important that appropriate strategies are identified to engage women with type 2 diabetes in planning their pregnancy and support them throughout their pregnancy journey and beyond.

Utilising the Socio‐Ecological Model, we identified personal, interpersonal and organisational factors that impact upon women's experiences throughout their pregnancy stages. On a personal level, women in the included studies reported feeling unable to have a ‘normal’ pregnancy due to their diabetes. This experience is echoed by previous studies in which women with diabetes and other chronic health conditions report the medicalisation of their pregnancy limiting their ability to experience the joy of a ‘normal’ pregnancy.^27^ This review has extended the understanding of women's experiences of elevated levels of worry and anxiety and has also identified a reduced sense of self‐efficacy. Such emotions have the potential to significantly negatively impact diabetes management, in terms of self‐monitoring and self‐care skills.^28^ It is essential that type 2 diabetes care during pregnancy is considerate of these issues and seeks to empower women, increase their skill and self‐efficacy and promote as ‘normal’ a pregnancy experience as possible. Furthermore, sufficient access to psychological support for women with type 2 diabetes throughout their pregnancy may be necessary to enable them to experience and enjoy positive aspects of their pregnancy and actively participate in their diabetes care.

The impact of family, friends and community perspectives on women's reproductive choices has been reported, highlighting the need to include women's social circle in discussions around pre‐pregnancy care.^8^ Indeed, women often rely upon information and advice from relatives or peers about managing their diabetes or their pregnancy.^29^ This was echoed in this review, as women reported a reliance on information from family and friends (interpersonal level). Additionally, some women in this review reported accessing online information. Consequently, healthcare providers may need to consider how they can utilise online and social media outlets to share evidence‐based information in an accessible manner for women with type 2 diabetes, with a focus on all stages from pre‐pregnancy to postpartum and future pregnancy planning. Lower health literacy is associated with less likelihood of accessing medical websites for health information and a greater likelihood of using social media, blogs and friends,^30^ contributing to inequalities in care. At a healthcare systems level, there is a need to consider how to target health literacy to ensure that women can make informed decisions about the trustworthiness of information sourced online and from family and friends, and to reduce inequalities.

For women living with type 2 diabetes, care is often disconnected and inconsistent, particularly in relation to their reproductive health.^8^, ^15^ This review aligns with such findings as, at an organisation level, women reported that type 2 diabetes care is often fragmented and inconsistent in relation to pregnancy. This can impact upon timeliness and continuity of care, leading to people feeling unsupported by the healthcare system and reducing engagement.^31^ Women in this review reported a desire for proactive discussions about pregnancy to be initiated by healthcare professionals from the point of type 2 diabetes diagnosis and for information and guidance to be explained in a clear and empathetic manner, rather than being delivered in a directive way. Previous research suggests that there are many factors influencing the interactions between healthcare professionals and women with type 2 diabetes, including lack of healthcare provider knowledge, biases and negative views held by providers, and time constraints during routine consultations.^8^ Unlike women with type 1 diabetes, in the United Kingdom, most women with type 2 diabetes are primarily cared for by generalist healthcare professionals in primary care rather than diabetes specialists, which may further impact upon the care provided. In a recent investigation into prescribing practices for women with type 2 diabetes aged 18 ‐ 45 years, we reported that 89% (*n* = 646) were not prescribed contraception and over one‐third (*n* = 269) of this cohort were prescribed medications not recommended for use in pregnancy.^32^ As well as skill and knowledge training, consideration must be given to how to address the external barriers that limit the opportunity for healthcare professionals to have positive and proactive pregnancy care discussions with women with type 2 diabetes. In the United Kingdom, the NHS's new programme, T2Day: Type 2 Diabetes in the Young, may help address some of these concerns, as it aims to enhance pregnancy awareness and promote medication review in people with type 2 diabetes under 40 years of age.^33^ This review has identified women experienced being removed from clinic recall lists during a pregnancy, thus hampering access to follow‐up care with their usual diabetes care providers in primary care post‐natally. This is an important finding and highlights the inadequate integration of services across the healthcare system for women with type 2 diabetes, and both a lack of clinic support and the limited understanding of postpartum care for this group.

### 4.1 Strengths and limitations

Despite the methodological rigour with which this review was conducted, there are limitations to acknowledge. Firstly, many of the studies included in this review were not exclusive to women with type 2 diabetes, as some also included the views of healthcare professionals, women with type 1 diabetes or gestational diabetes, and family members of women. As such, these studies were not designed to exclusively capture the nuances of the experiences of women with type 2 diabetes. The inclusion of these studies has deepened our understanding of the experiences of this group and only data explicitly from women with type 2 diabetes were included in this analysis and synthesis. It is important to consider the experiences of women with type 2 diabetes independently from other groups as their needs, experiences and contexts are likely to be different from women with other forms of diabetes. Therefore, future studies should consider concentrating on women with type 2 diabetes specifically.

Additionally, the number of studies included combined with the variety of study settings (7 different countries) meant that some reported experiences were unique to a particular population or setting, hence are not transferable to other women. The heterogeneity of this data means that the experiences of some women in the included studies may not be fully represented in this review, which reflects the common experiences of women with type 2 diabetes across settings and populations. Nonetheless, the range of perspectives included from women from diverse countries and settings is a strength of this review.

## 5 CONCLUSIONS

In conclusion, this review identified that despite the changing landscape of pregnancy among women with established diabetes, the experiences of women with type 2 diabetes have not improved in the last decade. Healthcare systems have failed to adjust to the needs of this group; consequently, women with type 2 diabetes report that healthcare providers do not proactively deliver information about pregnancy within their routine diabetes care. Until consideration is given to this group, the reproductive healthcare needs of women with type 2 diabetes will remain inherently biased against within the healthcare system.

## FUNDING INFORMATION

DS is supported by the National Institute for Health and Care Research (NIHR) through an NIHR Advanced Fellowship (NIHR302955) and the NIHR Southampton Biomedical Research Centre (NIHR203319).

CLM is funded by Diabetes UK through a Harry Keen Intermediate Clinical Fellowship (17/0005712) and the European Foundation for the Study of Diabetes—Novo Nordisk Foundation Future Leaders’ Award (NNF19SA058974).

SLW is supported by a grant from the Medical Research Council (UK) (MR/W003740/1).

## CONFLICT OF INTEREST STATEMENT

RF authored two of the included papers in this review.

## Supporting information


Data S1.

Data S2.
