# Supplementary material for: Experiences of women with type 2 diabetes during the pre‐pregnancy, pregnancy and postpartum periods: A systematic review of qualitative studies
Source: Diabet Med. 2025 Jul 3;42(9):e70094. doi: 10.1111/dme.70094 (PMC12352727; doi:10.1111/dme.70094)
Supplement: Supplementary file 2 — Data S2. [file DME-42-e70094-s001.docx]

| **Authors, Year**  **Supporting Information 2.** Overview of studies included in this review. | **Title** | **Data Collection Country** | **Population** | **Study Focus** | **Analysis Method** | **Key Themes** |
| --- | --- | --- | --- | --- | --- | --- |
| Britton et al., 2023^(19)^ | "A patient should not have to ask": Women's experiences of patient education about preconception care for type 2 diabetes | United States of America | 1) Women with type 2 diabetes, aged 18-40 years (n=32) | Pre-conception care | Content analysis | 1) Divergence between experiences and desires for pre-pregnancy care counselling  2) Why providers can’t wait for women to request pre-pregnancy care counselling  3) Perspectives on receiving information about pre-pregnancy care and pregnancy planning |
| Celik et al., 2023^(21)^ | ‘No one ever asks about something that actually is relevant to my life’: A qualitative study of diabetes and diabetes care experiences of young women with type 2 diabetes during their reproductive years | United Kingdom and Turkey | 1) Women with type 2 diabetes aged 18-45 years (n=36) | Experiences of women with Type 2 diabetes during reproductive years | Framework analysis | 1) Perception of self and identity as a young woman with Type 2 diabetes  2) Type 2 diabetes is not orientated to women’s needs  3) Components of improved care |
| Earle et al., 2017^(17)^ | Preconception care for women with type 1 or type 2 diabetes mellitus: a mixed-methods study exploring uptake of preconception care | United Kingdom | 1) White British or Pakistani women with type 1 (n=9) or type 2 diabetes (n=3) of childbearing age (18-45 years of age) | Pre-pregnancy care for women with diabetes | Thematic analysis | 1) Pregnancy choices and contraception  2) Enablers to preconception care  3) Barriers to preconception care  4) Communication with professional carers, patients or partners |
| Forde et al., 2020^(15)^ | A qualitative study exploring the factors that influence the uptake of pre-pregnancy care among women with Type 2 diabetes | United Kingdom | 1) Women with type 2 diabetes (n=30)  2) Healthcare professionals from primary, intermediate or secondary care (n=22) | Pre-pregnancy care for women with Type 2 diabetes | Framework analysis | 1) Lack of visibility of pre-pregnancy care in routine Type 2 diabetes care  2) Perceptions of Type 2 diabetes  3) Reproductive intention/potential  4) Communication on reproductive issues  5) Accessing pre-pregnancy care  6) Previous pre-pregnancy care experiences |
| Forde et al., 2021^(16)^ | An integrated pre-pregnancy care programme framework theoretically modelled from the perspectives of women with Type 2 diabetes and healthcare professionals | United Kingdom | 1) Women with type 2 diabetes of reproductive age (n=30)  2) Healthcare professionals from primary and secondary care (n=22) | Pre-pregnancy care for women with Type 2 diabetes | Framework analysis | 1) Enhancing understanding of reproductive needs among women and healthcare professionals  2) Constructing a more positive narrative for Type 2 diabetes and pregnancy  3) Enhancing the visibility of pre-pregnancy care  4) Integrated healthcare systems  5) Supportive technologies  6) A multi-modal approach |
| Johnson et al., 2020^(20)^ | A theoretical model of contraceptive decision-making and behaviour in diabetes: A qualitative application of the Health Belief Model | United States of America | 1) Women with type 1 (n=7) or type 2 diabetes (n=10), aged 18-50 years, premenopausal | Contraception care for women with diabetes | Iterative template coding method | 1) Perceived barriers and benefits to contraception use  2) Perceived seriousness of pregnancy  3) Perceived susceptibility to pregnancy risks  4) External cues to action  5) Internal cues to action  6) Self-efficacy  7) Modifying factors |
| Law et al., 2019^(24)^ | Influences on the decision to use contraception among Sarawakian women with diabetes: a qualitative exploration | Malaysia | 1) Women with type 2 diabetes aged over 18, and who were sexually active, and pre-menopausal (n=12) | Contraception care for women with diabetes | Thematic analysis | 1) Likelihood of becoming pregnancy  2) Desired family size  3) Personal health risks of getting pregnant  4) Social implications of getting pregnant  5) Opinion of significant others |
| Marshall et al., 2023^(18)^ | Experiences of Preconception Counselling among Pregnant Women with Preexisting Diabetes: Opportunities to Improve Patient-Centered Care | United States of America | 1) Pregnant women with preexisting diabetes:  type 1 (n=11), type 2 (n=10), unclassified (n=1) | Experiences of pre-conception counselling | Content analysis | 1) No standard version of counselling  2) Information provided during counselling typically focuses on the risk of diabetes and pregnancy  3) Patients seeking counselling felt their providers were generally supportive of their pregnancy desires, although there were a few exceptions  4) Insider knowledge  5) Desire for a different approach to counselling |
| Mukona et al., 2017^(25)^ | Barriers of Adherence and Possible Solutions to Nonadherence to Antidiabetic Therapy in Women with Diabetes in Pregnancy: Patients’ Perspective | Zimbabwe | 1) Pregnant women with diabetes:  type 1 (n=12), type 2 (n=10), GDM (n=6) | Barriers to antidiabetic therapy during pregnancy | Thematic analysis | 1) Barriers  2) Possible solutions to nonadherence to antidiabetic therapy |
| Sina et al., 2018^(22)^ | Development of an integrated, district-wide approach to pre-pregnancy management for women with pre-existing diabetes in a multi-ethnic population | Australia | 1) Women with type 1 or type 2 diabetes of Australian background (n=5)  2) Women with type 2 diabetes from cultural and linguistically diverse (CALD) and indigenous backgrounds (n=8)  3) Partners of CALD women (n=3) | Pre-pregnancy care for women with diabetes | Thematic analysis | 1) Early referrals for longer consultations  2) Barriers to contraception and pre-pregnancy management  3) Importance of awareness and access to educational resources  4) Partner support and investment in engagement |
| Sushko et al., 2023^(23)^ | Understanding the self-management experiences and support needs during pregnancy among women with pre-existing diabetes: a qualitative descriptive study | Canada | 1) Pregnant women with preexisting diabetes:  type 1 (n=6) type 2 (n=6) | Self-management and support needs during pregnancy | Content analysis | 1) Experiences of diabetes self-management in pregnancy  2) Patient-identified diabetes self-management support needs in pregnancy |
